# Unmet Cardiovascular Risk Beyond LDL-C: A Perspective on Managing Residual Cardiovascular Risk

**DOI:** 10.3390/ph19071071

**Published:** 2026-07-11

**Authors:** Kyriakos E. Kypreos, Anca Violeta Gafencu, Ourania Andreopoulou, Evangelia Zvintzou, Konstantinos Marinos, Victoria Mparnia, Maria Alemi, Bianca Sanziana Daraban, Marius Gabriel Multescu, Madalin Ghinea, Oana Mirancea

**Affiliations:** 1 Pharmacology Laboratory, Department of Medicine, University of Patras, 26500 Rio Achaias, Greece; andreop@upatras.gr (O.A.); liliazv@upatras.gr (E.Z.); kostasmarinos44@gmail.com (K.M.); victoriamparnia2@gmail.com (V.M.); alemimaria2@gmail.com (M.A.); 2 Gene Regulation and Molecular Therapies Laboratory, Institute of Cellular Biology and Pathology, “Nicolae Simionescu” of the Romanian Academy, 050568 Bucharest, Romania; anca.gafencu@icbp.ro (A.V.G.); sanziana.daraban@icbp.ro (B.S.D.); marius.multescu@icbp.ro (M.G.M.); madalin.ghinea@icbp.ro (M.G.); oana.mirancea@icbp.ro (O.M.); 3 School of Sciences, Department of Life Sciences, European University Cyprus, Egkomi, Nicosia 2404, Cyprus

**Keywords:** residual cardiovascular risk, atherosclerosis, apolipoprotein B, high-density lipoproteins, Lipoprotein(a), inflammation, dyslipidemias, mental health, risk factors

## Abstract

Despite major advances in lipid-lowering therapy and the widespread implementation of statins, ezetimibe, and PCSK9-targeted interventions, cardiovascular disease (CVD) remains the leading cause of morbidity and mortality worldwide. Intensive lowering of low-density lipoprotein cholesterol (LDL-C) has substantially reduced cardiovascular events; however, a considerable degree of residual cardiovascular risk persists even among patients achieving very low LDL-C levels. This observation has shifted contemporary cardiovascular research toward a broader understanding of atherosclerosis as a multifactorial disorder involving triglyceride-rich lipoproteins (TRLs), apolipoprotein B (APOB)-containing particles, dysfunctional high-density lipoproteins (HDLs), lipoprotein(a) [Lp(a)], endothelial dysfunction, and chronic vascular inflammation. Emerging evidence further suggests that psychological and behavioral factors, including specific personality traits and chronic psychosocial stress, may contribute independently to cardiovascular disease susceptibility and outcomes. The present review summarizes emerging risk factors associated with residual cardiovascular risk and highlights novel therapeutic strategies beyond LDL-C lowering. Collectively, current evidence supports a transition from a simplistic LDL-C-centric paradigm toward a multiple risk factor approach for atherosclerosis prevention and treatment.

## 1. Introduction

Atherosclerotic cardiovascular disease (ASCVD) is a chronic multifactorial disorder characterized by lipid accumulation, vascular inflammation, endothelial dysfunction, oxidative stress, and maladaptive immune responses within the arterial wall [1]. Since the mid-20th century, elevated plasma LDL-C has been recognized as a major causal risk factor for coronary heart disease (CHD), shaping decades of therapeutic development and clinical practice [2].

The historical foundations of modern lipidology emerged from seminal work performed during the 1940s and 1950s. Early studies by Cohn and colleagues, as well as Oncley, Scatchard, and Brown, identified major plasma lipoprotein fractions with α- and β-mobility, corresponding broadly to HDL and LDL particles, respectively [3,4]. Subsequently, the Framingham Heart Study established plasma lipoproteins as major predictors of cardiovascular disease and demonstrated the inverse association between HDL-C levels and CHD risk [2]. Over the following decades, large epidemiological and interventional studies unequivocally demonstrated that lowering LDL-C reduces cardiovascular events [5,6]. These findings led to the development of multiple therapeutic strategies aiming at lowering LDL-C, including statins, ezetimibe, bempedoic acid, PCSK9 monoclonal antibodies, small interfering RNA therapies, and emerging oral PCSK9 inhibitors [7,8,9,10,11].

Nevertheless, intensive LDL-C reduction does not abolish cardiovascular risk. Landmark clinical trials such as FOURIER revealed that a substantial residual risk persists even when LDL-C levels are reduced to historically unprecedented low levels [12,13]. This residual risk constitutes an unmet need that has prompted renewed interest in the additional atherogenic pathways that contribute to cardiovascular risk beyond LDL-C [1].

The contemporary understanding of residual cardiovascular risk now encompasses a multifactorial approach involving different biochemical factors, including elevated triglyceride-rich lipoproteins and remnant particles; increased numbers of APOB-containing lipoproteins; dysfunctional HDL particles; Lipoprotein(a); vascular inflammation; and psychological and behavioral factors, including personality traits that may independently influence cardiovascular susceptibility and outcomes. In contrast to plasma LDL-C levels, these factors are often less readily recognized, remain incompletely characterized, and are generally less well understood.

## 2. LDL-C Lowering: A Major Success Story with Important Limitations

The LDL hypothesis remains one of the most successful paradigms in preventive cardiology. Clinical and epidemiological studies consistently demonstrate that elevated LDL-C and non-HDL cholesterol are major causative factors in ASCVD development [14]. Current LDL-C-lowering therapies include statins, ezetimibe, bempedoic acid, anti-PCSK9 monoclonal antibodies, PCSK9 siRNA therapies [15], adnectin [16], and other emerging oral PCSK9 [17] and gene-editing based therapies [18,19]. The implementation of aggressive lipid-lowering guidelines has significantly reduced cardiovascular morbidity and mortality [20]. However, residual cardiovascular events continue to occur even among patients achieving very low LDL-C levels [10,21]. The FOURIER trial represented a milestone in this regard. Even though administration of evolocumab on top of maximum-tolerated statin and ezetimibe led to an unprecedented LDL-C lowering that produced major reductions in cardiovascular events, a substantial residual risk remained. These observations indicate that LDL-C reduction alone is insufficient to fully neutralize the complex biology of atherosclerosis [13]. The persistence of residual risk highlighted the existence of additional pathways contributing to plaque formation, vascular inflammation, thrombosis, and plaque instability.

## 3. Triglycerides and Triglyceride-Rich Lipoproteins

### 3.1. Epidemiological Evidence

Elevated plasma triglyceride (TG) levels are strongly associated with increased cardiovascular risk. Multiple epidemiological studies conducted over several decades have shown positive correlations between hypertriglyceridemia, atherosclerosis progression, and CHD incidence [22,23,24]. Importantly, high TG levels are frequently associated with increased oxidative stress, enhanced inflammation, endothelial dysfunction, pro-thrombotic state, adipose tissue dysfunction, and insulin resistance [25]. These findings support the concept that triglyceride-rich lipoproteins (TRLs) are not merely passive metabolic markers; they are active contributors to vascular pathology.

### 3.2. APOB-Containing Lipoproteins and Residual Risk

Triglycerides are highly lipophilic compounds, and as such, they are transported within an array of different APOB-containing particles: very low density lipoproteins (VLDLs), intermediate-density lipoproteins (IDLs), chylomicrons and chylomicron remnants, and LDL, including small, dense particles. Although plasma triglyceride concentration reflects lipid content, cardiovascular risk appears to correlate more closely with the number of APOB-containing particles [26].

A critical concept emerging from recent research is that two individuals with identical triglyceride levels may possess markedly different numbers of APOB-containing particles. Smaller, cholesterol-depleted, triglyceride-rich particles may dramatically increase particle number while maintaining similar triglyceride concentrations [26]. Moreover, these small, dense APOB particles, which are often as dense as HDL, are much more atherogenic than the much larger chylomicrons, VLDLs, and their remnants [27]. Even though earlier studies supported that plasma triglyceride levels constitute a modifying risk-factor for coronary heart disease [22,23,24], APOB burden, rather than triglyceride mass alone, may represent the more accurate measure of atherogenic risk [28].

### 3.3. Small, Dense APOB Lipoproteins

Small, dense LDL particles exhibit particularly high atherogenicity [28] due to several characteristics: enhanced arterial wall penetration, increased susceptibility to oxidation, reduced LDL receptor affinity, prolonged plasma residence time, and greater retention within the subendothelial space [29]. Even at physiologic triglyceride concentrations, elevated numbers of small, dense APOB-containing particles may substantially contribute to residual cardiovascular risk [30].

### 3.4. Pharmacological Approaches Targeting Triglyceride-Rich Lipoproteins

#### 3.4.1. Fibrates: Lessons Learned

Fibrates, including bezafibrate, ciprofibrate, fenofibrate, and gemfibrozil, were among the earliest pharmacological agents targeting hypertriglyceridemia. Although fibrates effectively lower triglycerides, clinical outcomes do not support a role for them in cardiovascular prevention and treatment [9]. This discrepancy between triglyceride-lowering and cardiovascular benefit highlighted the complexity of residual risk biology [31]. These findings contributed to an important conceptual transition: atheroprotection does not derive solely from lowering triglyceride mass.

#### 3.4.2. The Pemafibrate Story

Pemafibrate, a selective peroxisome proliferator-activated receptor alpha (PPARα) modulator, generated substantial enthusiasm due to potent triglyceride-lowering properties and favorable metabolic effects. However, major clinical trials failed to demonstrate expected cardiovascular benefits despite significant reductions in triglyceride levels [32]. These disappointing outcomes further emphasized that effective residual risk reduction extends beyond simple classical biomarkers and likely requires broader modulation of lipoprotein particle biology, inflammation, and vascular dysfunction [32]. The failure of pemafibrate to demonstrate cardiovascular benefit led to the final verdict that despite a major role of fibrates in preventing pancreatitis in acute severe hypertriglyceridemia, their use for cardiovascular risk mitigation in chronic moderate hypertriglyceridemia is futile.

### 3.5. Icosapent Ethyl and the High Purity EPA Paradigm

High-purity eicosapentaenoic acid (EPA) therapy has emerged as one of the most important recent advances in residual cardiovascular risk management. Icosapent ethyl (IPE), a highly purified EPA ethyl ester, became the first therapy of its class approved in Europe for cardiovascular risk reduction. Unlike mixed omega-3 fatty acid formulations, IPE demonstrated significant cardiovascular benefit in the REDUCE -IT clinical trial [33].

The benefits of IPE appear multifactorial and constitute a continuum that includes beneficial effects on lipid profiles, including reduction of triglyceride-rich particles and small dense APOB-containing particles; modulation of membrane lipid composition; anti-inflammatory effects; reduction in high-sensitivity C-reactive protein (hsCRP); modulation of inflammatory signaling pathways; anti-thrombotic effects; inhibition of platelet aggregation; improvement of endothelial function; antioxidant effects; optimized membrane fluidity; improved cellular signaling; and reduced oxidative stress [34]. Collectively, these pleiotropic effects, which are independent of starting plasma lipid levels or the extent of triglyceride reduction achieved, likely explain the approximately 25% reduction in cardiovascular death, myocardial infarction, and stroke observed in clinical studies. The success of IPE supports the concept that residual risk reduction requires interventions extending beyond simple triglyceride lowering.

### 3.6. Advanced Medicinal Products Targeting Apolipoprotein C3 and ANGPTL3

Apolipoprotein C3 (APOC3) and angiopoietin-like protein 3 (ANGPTL3) are key regulators of plasma triglyceride levels, primarily acting through the inhibition of lipoprotein lipase (LPL) activity. Both have recently emerged as pivotal pharmacological targets for the management of dyslipidemia and atherosclerotic cardiovascular disease (ASCVD) [35,36].

*APOC3 inhibitors:* Volanesorsen and olezarsen are antisense oligonucleotides that target hepatic APOC3 mRNA, thereby reducing plasma APOC3 protein levels. While volanesorsen was the first to be developed, its clinical utility is limited by a high incidence of drug-induced reduced platelet number; consequently, it did not receive FDA approval, though it is approved by the EMA for familial chylomicronemia syndrome. In contrast, olezarsen is a next-generation antisense oligonucleotide conjugated with a GalNAc moiety, which facilitates targeted hepatic delivery and improves the therapeutic index. Olezarsen has recently received both FDA and EMA approval for the management of familial chylomicronemia syndrome (FCS) and for the treatment of severe hypertriglyceridemia to reduce the risk of acute pancreatitis. Plozasiran is another antisense oligonucleotide targeting hepatic apoc3 mRNA indicated for the treatment of FCS [37].

While all three agents effectively lower plasma triglycerides, their ability to mitigate residual cardiovascular risk has not yet been confirmed in clinical outcomes trials, and they should not be used for this indication.

*ANGPTL3 inhibitors:* Evinacumab is a monoclonal antibody that targets ANGPTL3. Beyond its significant triglyceride-lowering effects, evinacumab has demonstrated the ability to reduce LDL-C levels by up to 30%, a unique benefit among contemporary triglyceride-lowering therapies. It is currently indicated as an adjunct to other lipid-lowering therapies for patients aged 1 year and older with homozygous familial hypercholesterolemia (HoFH) [38]. In addition to evinacumab, antisense oligonucleotides targeting ANGPTL3 mRNA are currently under development [36]. Similar to apoC3 mRNA-targeting strategies, the effectiveness of evinacumab in reducing cardiovascular risk has not been verified in large double-blinded, randomized clinical trials and should not be used for this indication.

## 4. High-Density Lipoprotein (HDL): From HDL-C Levels to HDL Functionality

### 4.1. Historical Perspective

HDL has historically been considered the “protective” lipoprotein. Early observations by John Gofman and subsequent epidemiological evidence demonstrated a strong inverse relationship between HDL-C levels and CHD risk [39,40]. The Framingham Heart Study [41] was the first to show unequivocally that the lower the levels of HDL-C in plasma, the higher the risk of CVD independently of other risk factors. This observation was later confirmed by many large-population studies, namely ARIC [42], INTERHEART [43], MRFIT [10], EPIC-Norfolk [44], and PROCAM [8], all of which consistently identified low HDL-C as a major independent cardiovascular risk factor [41]. Interestingly, recent data from the Framingham offspring study classify low plasma HDL-C levels as significant for coronary heart disease development as small, dense LDL particles and much more significant than plasma Lp(a) levels [27].

It is critical to distinguish between the genetic evidence of causality derived from Mendelian Randomization studies and the relative clinical burden of various lipid species derived from observational studies. Mendelian randomization studies have definitively established a causal role for elevated Lp(a) in the pathogenesis of atherosclerosis, identifying it as a high-priority therapeutic target for individuals with elevated genetic risk. However, this causal link should not overshadow the potent pro-atherogenic roles of small, dense LDL and dysfunctional HDL. While Lp(a) is often considered an independent risk factor, its relative contribution to residual cardiovascular risk compared to the burden of small, dense LDL and HDL may vary significantly especially across different patient phenotypes. While Lp(a) lowering is a primary target for specific at-risk populations, the clinical management of residual risk must remain holistic, addressing the cumulative pathogenic impact of all mediators, including small, dense LDL and dysfunctional HDL.

### 4.2. The HDL Paradox

These findings initially led to the belief that simply increasing HDL-C levels would produce cardiovascular benefit. Despite compelling epidemiological associations, clinical trials designed to pharmacologically raise HDL-C levels yielded disappointing results. Many HDL-targeted therapies produced substantial increases in HDL-C concentrations without corresponding reductions in cardiovascular events. This discrepancy became known as the “HDL paradox” [45]. To explain this observation, many potential explanations were proposed:

- HDL particles are structurally heterogeneous [46];
- Pharmacological HDL elevation does not improve HDL particle functionality [47];
- Large-scale manufacturing processes may alter recombinant HDL quality [48];
- Patient selection and trial design may influence outcomes [49].

These observations shifted scientific focus from HDL quantity toward HDL quality and functionality. Nevertheless, while HDL functionality is a promising therapeutic target, its clinical modulation remains complex due to the “HDL paradox” and the historic difficulties observed in previous large-scale clinical trials.

### 4.3. Functional Versus Dysfunctional HDL

Recent evidence strongly supports that not all lipoprotein particles with densities in the HDL density range are equally atheroprotective. Functional HDL particles exhibit efficient cholesterol efflux capacity, increased antioxidant activity, significant anti-inflammatory properties, vascular endothelial-layer protective effects, and anti-thrombotic actions [50]. In contrast, dysfunctional HDL particles may lose protective properties or even acquire pro-inflammatory characteristics [50].

### 4.4. Molecular Determinants of HDL Functionality

HDL functionality depends heavily on apolipoprotein composition and associated enzymes. Protective HDL-associated components include APOA-I, APOE, PON1, and S1P. Conversely, dysfunctional HDL may become enriched with serum amyloid A (SAA), oxidized phospholipids, myeloperoxidase (MPO), and APOCIII. As a result, changes in HDL proteomic composition may influence cholesterol efflux capacity and reverse cholesterol transport, antioxidant potential, anti-inflammatory signaling, and endothelial cell-layer functionality and repair mechanisms [50]. Experimental studies have demonstrated that HDL preparations differing in apolipoprotein content exhibit dramatically different biological properties.

### 4.5. The U-Shaped Relationship Between HDL-C and Cardiovascular Risk

Traditional models assumed a continuous near-linear inverse relationship between HDL-C levels and cardiovascular risk. However, more recent evidence suggests a U-shaped association. Specifically, very low HDL-C levels (<40 mg/dL) are associated with increased risk, intermediate HDL-C concentrations (40 to 70 mg/dL) appear optimal, 70–90 mg/dL appear borderline high (less optimal gray zone), and extremely high HDL-C levels (>90 mg/dL) may be associated with significantly increased risk [51,52]. This concept may partially explain failures of CETP inhibitor trials, in which HDL-C levels often increased to extraordinarily high concentrations [49]. Rather than indiscriminate HDL-C elevation, future strategies may require selective enhancement of HDL functionality while maintaining physiologic HDL concentrations [45].

## 5. Lipoprotein(a): An Emerging Major Contributor to Residual Risk

### 5.1. Historical Background

Lipoprotein(a) [Lp(a)] has emerged as a major genetically determined cardiovascular risk factor. Lp(a) was first described by Kåre Berg in 1963 as a heritable plasma trait [53]. Subsequent molecular studies revealed that Lp(a) consists of an LDL-like particle covalently linked to apolipoprotein(a), a protein exhibiting structural homology to plasminogen. Mendelian randomization studies later established a causal relationship between elevated Lp(a) and ASCVD [54].

### 5.2. Pathophysiological Mechanisms

Lp(a) contributes to atherogenesis through multiple pathways [54]:

- Promotion of cholesterol deposition [55];
- Oxidized phospholipid transport [56];
- Endothelial dysfunction [57];
- Vascular inflammation [58];
- Prothrombotic activity due to plasminogen homology at extreme Lp(a) levels [59].

Currently, Lp(a) is considered a biomarker associated with increased atherosclerosis risk, and plasma levels should be <50 mg/dL.

### 5.3. Emerging Therapies

Extrapolating the association of elevated plasma Lp(a) levels with atherosclerosis, novel therapeutic approaches targeting elevated Lp(a) are currently under phase III clinical trials. They include the following:

- Antisense oligonucleotides, with pelacarsen serving as the primary representative, act through RNase H-mediated degradation of apolipoprotein(a) mRNA in the nucleus. Clinical data indicate that monthly administration of pelacarsen results in a reduction of plasma Lp(a) levels of up to 67% [60].
- siRNA-based therapies that utilize the RNA-induced silencing complex (RISC) in the cytoplasm to achieve highly specific and catalytic cleavage of apolipoprotein(a) mRNA. Notable representatives include olpasiran, which can reduce plasma Lp(a) levels by up to 95%, and lepodisiran, which can achieve reductions of up to 96% when administered bi-annually [61,62].
- Small-molecule APO(a)-directed approaches: Muvalaplin that can lower plasma Lp(a) levels up to 65% when administered orally daily [63].

Conventional lipid-lowering therapies have produced mixed effects on Lp(a). Niacin has been shown to reduce Lp(a) levels by more than 30% [64]; however, evidence from AIM-HIGH (ClinicalTrials.gov: NCT00120289) [65] and the HPS2-THRIVE (ClinicalTrials.gov: NCT00461630) [66] showed no cardiovascular benefit. On the other hand, statins increase Lp(a) levels by up to 25% [67]. Given the undisputed benefit of statins in reducing cardiovascular risk, the statin-associated increase in Lp(a) levels raises an important question regarding the true contribution of Lp(a) lowering to overall cardiovascular risk reduction. Adding to this, recent observational data from the Offspring Framingham Heart Study indicate that Lp(a) is a CVD risk factor, but its significance appears much lower than other risk factors, such as elevated small, dense LDL-C and reduced HDL-C levels [27].

## 6. Inflammation as a Central Driver of Atherosclerosis

Atherosclerosis has evolved from a historical conceptualization as a passive lipid-storage disease into the current understanding of a chronic, immune-mediated inflammatory disorder [68]. The initiation and progression of atherosclerotic plaques are fundamentally driven by the recruitment and activation of innate immune cells. Monocytes are recruited to the arterial intima, where they differentiate into macrophages, subsequently evolving into foam cells following the ingestion of modified lipoproteins [68]. These macrophages are not merely passive reservoirs for lipid accumulation; they act as primary effectors of the plaque microenvironment, coordinating complex cellular responses that drive lesion expansion and instability. The persistence of these cells within the plaque, maintained by localized proliferation and the recruitment of fresh progenitors, perpetuates a self-reinforcing cycle of chronic inflammation [69]. Central to the inflammatory landscape of the plaque is the orchestration of potent signaling cascades. The NF-κB pathway serves as a master transcriptional regulator, facilitating the expression of pro-inflammatory cytokines, adhesion molecules, and chemokines that sustain the inflammatory phenotype [70]. Furthermore, the activation of the HMGB1/RAGE/NLRP3 inflammasome axis represents a critical bridge between cell stress and the propagation of inflammation. The assembly of the NLRP3 inflammasome facilitates the maturation and release of IL-1β and IL-18, cytokines that amplify local tissue injury and promote the recruitment of further inflammatory cell populations [71]. This molecular crosstalk is instrumental in the transition of plaques toward vulnerable phenotypes, characterized by thinning of the fibrous cap and an enlarged necrotic core. Additional contributors to vascular inflammation are neutrophil extracellular traps (NETs) [72], which act as potent pro-inflammatory and pro-thrombotic drivers within the atherosclerotic plaque by facilitating macrophage activation, enhancing necrotic core formation, and promoting platelet aggregation. Moreover, immunometabolic reprogramming of atherosclerosis infiltrating macrophages [73] involves a critical shift toward aerobic glycolysis, which sustains the chronic inflammatory response while impairing the clearance of cellular debris, thereby accelerating plaque progression and vulnerability.

The transition of these mechanistic insights into clinical practice is a primary objective of modern vascular medicine. Identifying and modulating these specific molecular targets—rather than solely focusing on LDL-cholesterol reduction—offers a potential avenue for addressing the unmet clinical need of residual cardiovascular risk. By elucidating the complex interactions between cellular drivers and molecular pathways, current research continues to highlight the potential for novel pharmacological approaches to refine the management of cardiovascular disease. Below, we prioritize those strategies that have reached clinical implementation with immediate relevance to patient management.

### 6.1. The CANTOS Trial

The CANTOS trial represented a landmark proof-of-concept study demonstrating that anti-inflammatory therapy can reduce cardiovascular events independently of lipid lowering [74]. Canakinumab, a monoclonal antibody targeting interleukin-1β (IL-1β), significantly reduced recurrent cardiovascular events in patients with a prior myocardial infarction and a high-sensitivity C-reactive protein level of ≥2 mg/L, without altering lipid levels. These findings provided strong direct evidence that inflammation itself represents a therapeutic target in ASCVD.

### 6.2. IL-6 Targeting and Ziltivekimab

Interleukin-6 (IL-6) occupies an upstream position within the inflammatory cascade and drives hepatic CRP production. Ziltivekimab, a fully humanized monoclonal antibody targeting IL-6, demonstrated potent reductions in hsCRP and thrombosis-related biomarkers in the RESCUE trial [75]. The ongoing ZEUS trial is evaluating whether IL-6 inhibition can reduce cardiovascular events among high-risk patients with chronic kidney disease and systemic inflammation [76]. Collectively, these studies suggest that anti-inflammatory therapy may become a major component of future cardiovascular prevention.

## 7. Infections and Residual Cardiovascular Risk

Recent evidence underscores the role of acute and chronic infections as significant drivers of residual cardiovascular risk [77]. Specifically, infections severe enough to require hospital treatment were associated with a significantly increased short-term risk of major cardiovascular disease events and a slightly increased long-term risk [77]. Specifically, respiratory infections such as the respiratory syncytial virus (RSV) contribute to a substantial increase in cardiovascular disease (CVD) burden, resulting in an excess risk of cardiovascular events over a one-year period that is comparable in magnitude to that observed with influenza infection. Furthermore, other viral infections such as SARS-CoV-2, HIV, hepatitis C and herpes zoster virus have been identified as a contributor to long-term residual cardiovascular risk, with its manifestation linked to an increased incidence of major adverse cardiovascular events [78,79]. Clinicians should remain cognizant that hospitalization for a severe infection is associated with an elevated risk of major cardiovascular events. Consequently, where applicable, vaccinations should be incorporated into clinical practice as a preventive strategy to mitigate this excess cardiovascular risk.

## 8. The Psychological Component—Personality Traits and Residual Cardiovascular Risk

In addition to advances in pharmacotherapy, advances in the understanding of the psychological component in residual cardiovascular risk is gaining increasing attention, as psychosocial characteristics are independent risk factors that contribute to significant residual risk, even after optimal adjustment for standard risk factors [80]. The recent literature highlights that factors such as depression, anxiety, and personality traits are associated with increased mortality and major adverse cardiac events (MACEs) through complex biological and behavioral pathways [81,82]. Specifically, chronic activation of the sympathetic nervous system and dysfunction of the hypothalamic–pituitary–adrenal (HPA) axis have been implicated in endothelial dysfunction, vascular inflammation, and adverse cardiovascular remodeling, while psychological distress may also compromise medication adherence and the adoption of healthy lifestyle behaviors [80,83]. Therefore, it is becoming increasingly evident that cardiovascular disease should not be addressed as an isolated entity but rather as a manifestation of a complex biopsychosocial condition in which psychological, cardiovascular, and biochemical factors are closely interconnected [84]. The adoption of a “Psycho-Cardiology” approach that incorporates systematic screening with tools such as the WHO-5 or the PHQ-9 and personalized psychological interventions (stepped-care models) is recommended as an essential component of the holistic management of patients with cardiovascular disease, with the potential to reduce residual morbidity not adequately addressed by conventional therapeutic strategies [80,85].

## 9. Residual Cardiovascular Risk: Multiple Factor Approach

The modern concept of residual cardiovascular risk extends far beyond traditional LDL-C levels. Current evidence supports a multifactorial model involving:

- APOB particle number;
- Triglyceride-rich lipoproteins;
- Small, dense LDL particles;
- Dysfunctional HDL;
- Lipoprotein(a);
- Vascular inflammation;
- Oxidative stress;
- Endothelial dysfunction;
- Pro-thrombotic signaling;
- Psychological and behavioral factors.

**Figure 1:**
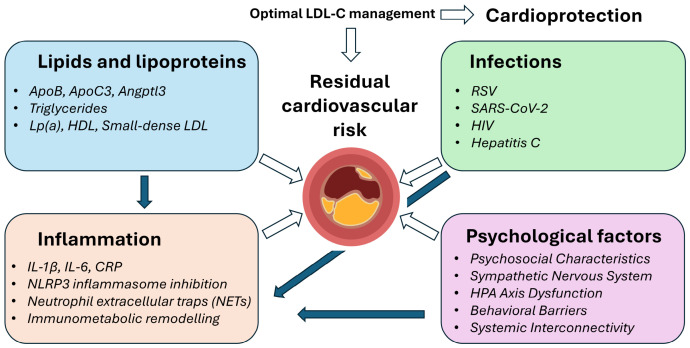
The multifactorial nature of residual cardiovascular risk in the era of optimal LDL-cholesterol management. While rigorous reduction of low-density lipoprotein cholesterol (LDL-C) remains the foundational approach for cardioprotection, it is insufficient to eliminate all cardiovascular events. Residual cardiovascular risk persists due to the synergistic interplay of multiple, independent pathogenic drivers. This residual risk is primarily shaped by four interrelated domains: (1) lipids and lipoproteins, encompassing the pro-atherogenic burden of Apolipoprotein B (ApoB)-containing particles, triglyceride-rich lipoproteins (TRLs), small/dense LDL, Lipoprotein(a) [Lp(a)], and dysfunctional high-density lipoprotein (HDL) particles; (2) inflammation, a central driver of plaque instability, is mediated by cytokine signaling (IL-1β, IL-6, and CRP), NLRP3 inflammasome activation, the formation of neutrophil extracellular traps (NETs), and immunometabolic reprogramming of arterial macrophages; (3) infections, including acute respiratory viruses (e.g., RSV and SARS-CoV-2) and chronic conditions (HIV and Hepatitis C), which significantly elevate the short- and long-term risk of major adverse cardiovascular events; and (4) psychological factors, where psychosocial characteristics, sympathetic nervous system hyperactivity, hypothalamic–pituitary–adrenal (HPA) axis dysfunction, and behavioral barriers collectively exacerbate cardiovascular risk through both systemic biological stress and impaired therapeutic adherence. Comprehensive clinical pharmacology strategies must transition from a simplistic, LDL-C-centric paradigm toward a personalized, multifactorial framework that targets these diverse pathogenic contributors to effectively mitigate residual risk. White arrows indicate the direct causality of these factors, and blue arrows indicate their interconnection. The center of the illustration, where all white arrows converge, represents a cross-section of an artery with established atherosclerotic plaque.

Despite that LDL-C reduction has revolutionized ASCVD management, leading to significant risk mitigation, these risk factors act as independent contributors to the disease, conferring a synergistic amplifying effect on cardiovascular risk. For example, hypertriglyceridemia promotes the formation of small, dense LDL particles; inflammation induces HDL dysfunction; oxidative stress modifies lipoprotein composition; and endothelial injury amplifies inflammatory signaling. Furthermore, accumulating evidence suggests that psychological factors, including chronic stress, depression, anxiety, and specific personality traits, may contribute independently to residual cardiovascular risk through both biological and behavioral mechanisms [80]. Therefore, future therapeutic approaches will likely require personalized, multi-risk-factor strategies targeting both established metabolic pathways and emerging non-traditional determinants of cardiovascular risk, potentially extending beyond conventional clinical management paradigms (Figure 1).

## 10. Future Perspectives

The landscape of cardiovascular prevention is shifting rapidly. Future risk stratification and therapeutic frameworks are poised to expand beyond conventional metrics, such as low-density lipoprotein cholesterol (LDL-C), to integrate advanced, precise lipidomic and inflammatory markers and therapeutic targets:

- Plasma apolipoprotein B (ApoB): Prioritizing plasma ApoB levels, with a specific focus on the burden imposed by highly atherogenic, small, dense lipoprotein particles [86].
- Advanced lipoprotein profiling: Focusing on detailed particle characterization, including subspecies profiling of small/dense lipoproteins, triglyceride-rich lipoproteins, LDL, and HDL [27].
- HDL functionality: Implementing clinical-grade HDL functional assays, encompassing comprehensive proteomic and lipidomic mapping, alongside targeted therapies engineered to restore or enhance HDL particle quality [49].
- Inflammatory burden assessment: Systematically tracking validated inflammatory biomarkers to better capture and mitigate residual inflammatory risk [87].

The success of siRNA, antisense, and gene-editing technologies targeting PCSK9 and Lp(a) opens the possibility for highly selective modulation of previously “undruggable” pathways. Moreover, to address the psychological component of residual risk, it is important that cardiologists are aware of recognizing patients who need psychological support and refer them early to mental health care professionals [80].

## 11. Conclusions

The reduction of LDL-C remains the cornerstone of cardiovascular prevention; however, substantial residual cardiovascular risk persists despite aggressive LDL-C-lowering therapies. Contemporary clinical evidence demonstrates that this residual risk constitutes a critical unmet need driven by an intricate network of non-traditional pathways, including triglyceride-rich ApoB-containing lipoproteins, lipoprotein(a), chronic vascular inflammation, and psychosocial stressors.

The clinical failure of early-generation HDL-raising and generic triglyceride-lowering therapies highlights the limitations of simplistic, biomarker-centric treatment paradigms. Instead, optimized management demands a nuanced, mechanism-driven approach. Translational pharmacology is rapidly addressing these gaps through targeted therapeutics—such as antisense oligonucleotides and siRNA therapies targeting $Lp\left(a\right)$ and APOC3 mRNA, as well as targeted anti-inflammatory agents—which transition the field from mere biomarker reduction to the modulation of specific pathogenic pathways. Furthermore, integrating biochemical intervention with targeted psychosocial support acknowledges the complex, multifactorial nature of cardiovascular disease management. Ultimately, mitigating residual cardiovascular risk requires a shift from a strictly cholesterol-centric model toward a multifactorial, personalized, clinical pharmacology framework.

Future cardiovascular prevention strategies will likely depend on personalized approaches that combine lipid modulation, inflammatory control, functional assessment of lipoprotein biology, and psychological support, where needed. As our understanding of residual cardiovascular risk continues to evolve, the field is progressively shifting from an LDL cholesterol-centric view of atherosclerosis toward a more comprehensive, multifactorial disease, requiring pharmacotherapy toward multiple contributing factors.

## Data Availability

No new data were created or analyzed in this study. Data sharing is not applicable to this article.

## Abbreviations

The following abbreviations are used in this manuscript:

ANGPTL3	Angiopoietin-like protein 3
APOA-I	Apolipoprotein A-I
APOB	Apolipoprotein B
APOC3	Apolipoprotein C3
APOE	Apolipoprotein E
ARIC	Atherosclerosis Risk in Communities
ASCVD	Atherosclerotic cardiovascular disease
CETP	Cholesteryl ester transfer protein
CHD	Coronary heart disease
CVD	Cardiovascular disease
EPA	Eicosapentaenoic acid
EPIC	European Prospective Investigation into Cancer and Nutrition
HPA	Hypothalamic–pituitary–adrenal
HDL	High-density lipoprotein
HDL-C	High-density lipoprotein cholesterol
hsCRP	High-sensitivity C-reactive protein
IDL	Intermediate-density lipoprotein
IL-1β	Interleukin-1β
IL-6	Interleukin-6
IPE	Icosapent ethyl
LDL	Low-density lipoprotein
LDL-C	Low-density lipoprotein cholesterol
Lp(a)	Lipoprotein(a)
MACE	Major adverse cardiac events
MPO	Myeloperoxidase
MRFIT	Multiple Risk Factor Intervention Trial
PHQ-9	Patient Health Questionnaire-9
PCSK9	Proprotein convertase subtilisin/kexin type 9
PON1	Paraoxonase 1
PPARα	Peroxisome proliferator-activated receptor alpha
PROCAM	Prospective Cardiovascular Münster
S1P	Sphingosine-1-phosphate
SAA	Serum amyloid A
sdLDL-C	Small, dense low-density lipoprotein cholesterol
siRNA	Small interfering RNA
TG	Triglyceride
TRLs	Triglyceride-rich lipoproteins
VLDL	Very low density lipoprotein
WHO-5	World Health Organization-5 Well-Being Index
